# Ectoine from Bacterial and Algal Origin Is a Compatible Solute in Microalgae

**DOI:** 10.3390/md18010042

**Published:** 2020-01-06

**Authors:** Simona Fenizia, Kathleen Thume, Marino Wirgenings, Georg Pohnert

**Affiliations:** 1 Institute for Inorganic and Analytical Chemistry, Bioorganic Analytics, Friedrich Schiller University, Lessingstrasse 8, D-07743 Jena, Germany; simona.fenizia@uni-jena.de (S.F.); Kathleen.thume@uni-jena.de (K.T.); Marino.wirgenings@uni-jena.de (M.W.); 2 Max Planck Institute for Chemical Ecology, Hans-Knöll-Straße 8, D-07745 Jena, Germany

**Keywords:** ectoine, osmoadaptation, compatible solutes, phytoplankton, LC/MS analysis, osmoregulation, diatoms, DMSP

## Abstract

Osmoregulation in phytoplankton is attributed to several highly polar low-molecular-weight metabolites. A widely accepted model considers dimethylsulfoniopropionate (DMSP) as the most important and abundant osmotically active metabolite. Using an optimized procedure for the extraction and detection of highly polar metabolites, we expand the group of phytoplankton osmolytes by identifying ectoine in several microalgae. Ectoine is known as a bacterial compatible solute, but, to the best of our knowledge, was never considered as a phytoplankton-derived product. Given the ability of microalgae to take up zwitterions, such as DMSP, we tested the hypothesis that the algal ectoine is derived from associated bacteria. We therefore analyzed methanol extracts of xenic and axenic cultures of two different species of microalgae and could detect elevated concentrations of ectoine in those that harbor associated bacteria. However, also microalgae without an associated microbiome contain ectoine in smaller amounts, pointing towards a dual origin of this metabolite in the algae from their own biosynthesis as well as from uptake. We also tested the role of ectoine in the osmoadaptation of microalgae. In the model diatoms *Thalassiosira weissflogii* and *Phaeodactylum tricornutum*, elevated amounts of ectoine were found when cultivated in seawater with salinities of 50 PSU compared to the standard culture conditions of 35 PSU. Therefore, we add ectoine to the family of osmoadaptive metabolites in phytoplankton and prove a new, potentially synergistic metabolic interplay of bacteria and algae.

## 1. Introduction

Diatoms are photosynthetic unicellular algae, responsible for 20% of global carbon fixation and 40% of marine primary production. They are important producers of zwitterionic metabolites, a class of small organic compounds that has central osmoregulatory, antioxidant, and cryoprotectant functions [1,2]. Dimethylsulfoniopropionate (DMSP) is the main representative of this class of molecules: it is a sulfur-containing metabolite reaching high cellular concentrations in marine algae [1,3]. Marine bacteria and algae metabolize DMSP into the correspondent volatile compound dimethylsulfide (DMS), contributing to the flux of sulfur from the hydrosphere to the atmosphere [3]. Other sulfur-containing metabolites, like dimethylsulfonioacetate (DMSA), gonyol, and the most recently identified dimethylsulfoxonium propionate (DMSOP); as well as nitrogen-containing metabolites, like glycine betaine (GBT), homarine, and trigonelline, have been discovered and studied in phytoplankton as well [2,4]. These metabolites are classified as “compatible solutes”, organic water-soluble compounds accumulated by micro-organisms either by de novo synthesis or by uptake from the surrounding environment. They protect the cells from stress factors, such as environmental changes in temperature, pH, and salinity [5].

Salinity is an environmental master factor for marine organisms, affecting their distribution, reproduction, and behavior [6]. To cope with changing salinity in the oceans, marine algae synthesize, take up, and accumulate compatible solutes to keep physiological levels of cellular hydration and turgor [7,8]. The synthesis of these compounds is energetically costly, requiring assimilation and reduction of sulfate or nitrate [1]. Since nitrogen sources are often limiting in marine habitats, sulfur-containing osmolytes are more abundant compared to N-containing counterparts. It can thus be argued that the high production of DMS can be linked to the preference of N-limited phytoplankton communities that use DMSP instead of, for example, glycine betaine [9].

In the last years, many organic osmolytes produced by microalgae were identified and their contribution to the osmoadaptation and osmoregulation of marine ecosystems pointed out [2]. A complex picture emerges, showing that the common DMSP/DMS concept represents a massive oversimplification. Osmoadaptation seems to depend on a plethora of metabolites of which only few have been structurally elucidated [2]. Major progress in the field is enabled by the development of direct analytical methods of zwitterions. Spielmeyer and Pohnert [10,11] introduced an ultra performance liquid chromatography (UPLC) method for separation and direct determination of DMSP, GBT, and other zwitterionic metabolites produced by marine phytoplankton. A further liquid chromatography/mass spectrometry (LC/MS) survey of microalgal extracts revealed that the structure and function of several highly polar, most likely zwitterionic small metabolites are still uncharacterized [4]. This study aims to identify such uncharacterized metabolites and to investigate their regulation under osmotic stress. We took advantage of a refined analytical method using high-resolution mass spectrometry (based on [7] and [10]) to analyze, qualitatively and quantitatively, unknown components in the “zwittermetabolome” of the diatoms *Thalassiosira weissflogii* and *Phaeodactylum tricornutum*. These two diatoms were selected because they are well-established model organisms for phytoplankton studies. *P. tricornutum* is the first pennate diatom for which the complete genome is known [12]. *T. weissflogii* is one of the few diatoms identified that does not produce quantifiable amounts of DMSP [7]. The alga has previously been used to study acclimation to hyposalinity with a reported detailed transcriptomic and physiologic survey of the response to this stress factor [13]. We observed that osmoadaptation is achieved in both algae by adjustment of intracellular concentrations of different zwitterionic compounds, including the novel algal mediator, ectoine. This metabolite can thus be counted among the phytoplankton-compatible solutes. Moreover, direct uptake of bacteria-derived ectoine by the diatom *T. weissflogii* was detected in a mechanism that represents an economic strategy to acquire essential metabolites instead of their de novo production.

## 2. Results and Discussion

To identify novel zwitterionic metabolites involved in the osmoadaptation of marine organisms, we analyzed the endometabolome of the diatoms *Thalassiosira weissflogii* and *Phaeodactylum tricornutum* using ultra-high-pressure liquid chromatography high-resolution mass spectrometry (UHPLC–HRMS). Algae were grown at standard culture conditions of 35 Practical Salinity Units (PSU, g NaCl kg^−1^ sea water) and under increased salinity of 50 PSU, where the growth of *T. weissflogii* still follows a standard growth curve with slightly reduced cell counts [14]. The selected salinities span the range of saline water and allow the linking of our results to previous studies on osmoregulation of algae [14,15,16]. We detected the production of several known, but also of structurally unassigned, highly polar low-molecular-weight metabolites under the different salinity concentrations.

### 2.1. Thalassiosira weissflogii

**Figure 1:**
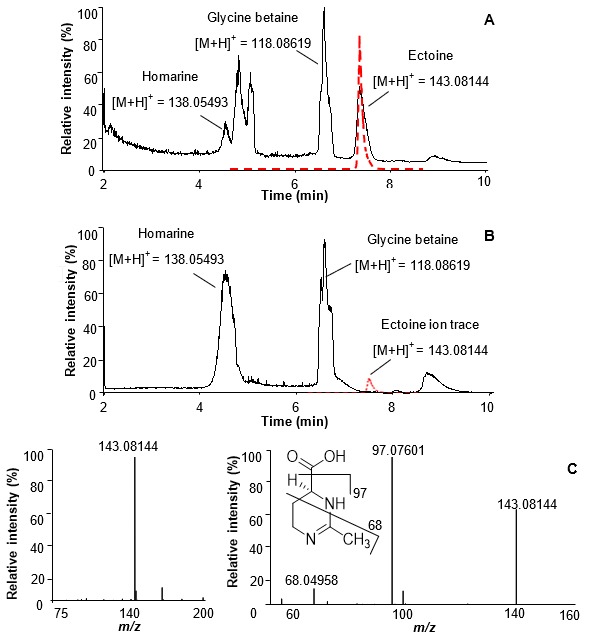
Chromatographic separation of zwitterionic metabolites in *Thalassiosira weissflogii* RCC76 (35 PSU xenic, (**A**)) and CCMP1336 (35 PSU axenic, (**B**)) using UHPLC–HRMS. The red line in (**A**) represents the UHPLC–HRMS monitoring of the ion trace *m*/*z* = 143.08144 ± 0.0005 after addition of a synthetic ectoine standard, peaks at 5 and 5.2 min are contaminants. The dashed red line in (**B**) depicts the ion trace of *m*/*z* = 143.08144 ± 0.0005. (**C**), electrospray ionization (ESI) MS and tandem mass spectrometry (MS/MS) of ectoine with characteristic fragments depicted. The identity of the metabolites glycine betaine and homarine were assigned using synthetic standards according to previous studies [2].

Figure 1 shows UHPLC–HRMS chromatographic profiles obtained from extracts of xenic and axenic *T. weissflogii* (respective strains used were RCC76 and CCMP1336). This diatom does not produce any quantifiable amount of DMSP [7], the main zwitterionic metabolite produced by many other planktonic algae [4]. In the xenic algal culture, we detected three major zwitterionic metabolites, glycine betaine and homarine, previously identified in diatoms, as well as an unknown metabolite [9].

The identity of glycine betaine was verified by co-injection with a commercially available standard. This metabolite is the nitrogen-containing analog of DMSP and is produced by marine phytoplankton dependent on nitrogen availability [17]. Homarine was synthesized in our laboratory according to a published procedure [2,4], and co-injection with the algal extract confirmed its identity (see experimental section for details). Homarine is a nitrogen-containing zwitterion, but, to the best of our knowledge, it was described only as a minor component in the microalgae *Emiliania huxleyi* and *Platymonas subcordiformis* [2,4,18]. Our data shows that it is a major zwitterionic metabolite in *T. weissflogii*.

In addition to the signals of these two metabolites known from microalgae, the chromatogram and the HRMS spectrum in positive ionization mode shows a peak with *m*/*z* of the [M + H]^+^ = 143.08144, corresponding to the molecular formula C_6_H_11_O_2_N_2_ (Figure 1). A fragment ion *m*/*z* 97.07601 was detected by tandem mass spectrometry (MS/MS) and was attributed to the loss of a carboxylic group. An ion at *m*/*z* 68.04958 indicated a loss of C_3_H_4_O_2_N (Figure 1C). Based on the mass spectrometric data, by comparison with databases (METLIN, MassBank of North America, MetaboLights), and by using bioinformatics tools (Sirius v4.0.1, CSI:FingerID), the signal was tentatively assigned to ectoine. The identity was verified by co-injection of the algal extract with a commercially available standard. Coelution and matching MS/MS data of the analyte and the standard proves the identity of this metabolite as ectoine (Figure 1).

Ectoine, 2-methyl-1,4,5,6-tetrahydropyrimidine-4-carboxylic acid, was initially isolated and identified from the halophilic phototrophic sulfur bacterium *Halorhodospira halochloris*. This metabolite has protective and osmoregulatory functions during adaptation to salinity changes and is therefore classified as bacterial compatible solute [19,20]. Thanks to its hydrophilicity, ectoine has additional protective properties, stabilizing bacterial cells against different kinds of stress like UV radiation and cytotoxins [21]. Besides in bacteria [21,22], ectoine is also found in halophilic ciliates including *Schmidingerothrix salinarum* [23,24], and the halophilic bacterivorous nanoflagellate *Halocafeteria seosinensis* [25]. Some members of Archaea acquired ectoine genes through horizontal gene transfer to cope with a high-salinity environment [26]. Based on gene expression patterns of bacteria in coculture with the diatom *T. pseudonana*, Landa et al. postulated that the alga might provide ectoine to the bacterium *Rugeria pomeroyi* [27]. However, to the best of our knowledge, the production of this metabolite by diatoms has never been reported.

**Figure 2:**
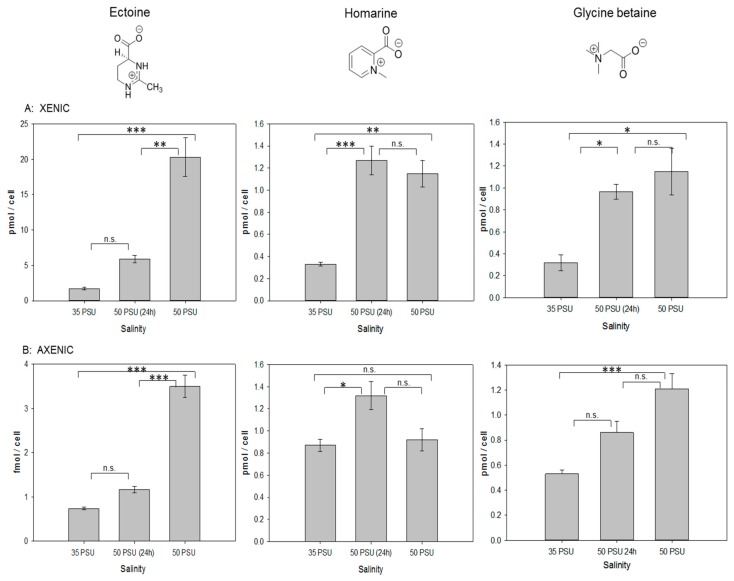
Comparison of intracellular amounts of ectoine and homarine in xenic (**A**) and axenic (**B**) cultures of *T. weissflogii*. The value of 35 PSU indicates that cultures were maintained constantly at this salinity, 50 PSU (24 h) indicates that cultures grown at 35 PSU were transferred into medium of 50 PSU and analyzed 24 h after transfer, and 50 PSU indicates cultures that were grown for two generations at this elevated salinity. Concentrations are normalized per cell, error bars represent standard deviation (biological replicates, *N* = 4). Statistical analysis is based on One-Way ANOVA with a Tukey Test for multiple comparison procedures. * *P* ≤ 0.05, ** *P* ≤ 0.01, *** *P* ≤ 0.001.

The fact that axenic diatoms produce low, but significant amounts of ectoine (Figure 2) prompted us to mine the genome of the fully sequenced diatom *Phaeodactylum tricornutum* for genes involved in ectoine biosynthesis. In bacteria, ectoine is synthesized by three proteins: EctA, EctB, and EctC. We found homologues of EctA (XP_002181681.1, E value 0.15) and EctB (XP_002185537.1, E value 6 × 10^−50^) that provide the central intermediate Nγ-acetyl-2,4-diaminobutyrate but not for EctC. If the last step, namely, the condensation to ectoine is catalyzed by an EctC protein with low homology or if another condensating enzyme activity is involved remains still open.

### 2.2. Response to Osmotic Stress of T. weissflogii

To verify osmoregulatory functions of ectoine in the diatom, cells were grown at low (35 PSU) and high (50 PSU) salinity. The higher salinity resulted in reduced cell counts, indicating salinity stress as already observed previously [15]. For short-term salinity stress experiments, cells were grown at 35 PSU to the late exponential phase and transferred to 50 PSU. Analysis of these cells was done 24 h after transfer. Tests were performed on both xenic and axenic cultures of *T. weissflogii* (Figure 2). Xenic cultures were harvested by filtration on GF/C filters, which removes the most part of the bacteria. We verified the removal of bacteria by flow cytometry of the filtrate and found no significant difference between filtrate and culture (filtrate, 17.550 ± 19 cells µL^−1^; xenic culture, 17.5120 ± 31 cells µL^−1^). We also observed remaining bacteria caught in the filter that might contribute to the detected osmolytes. This fraction can, however, be only minor given the high filtration success.

Quantification of ectoine revealed that the cellular content in *T. weissflogii* increases during osmotic stress conditions, in particular in xenic cultures, where this molecule is the dominant detected osmolyte (Figure 2). When a short-term stress was introduced, ectoine concentration increased by 3.5-fold compared to the concentration under 35 PSU conditions. Long-term salinity stress led to an over 11-fold increase of ectoine. In axenic cultures, the intracellular concentration of ectoine was more than three orders of magnitude lower compared to xenic ones. In the cultures lacking the associated bacteria, the ectoine content increased 1.6-fold during the short-term salinity stress and 4.8-fold during the long-term stress (Figure 2B). Cell size and volume determined by light microscopy did not change in *T. weissflogii* under osmotic stress conditions (Table 1). Therefore, changes in intracellular amounts of the osmoprotectants also reflect changes of intracellular concentration. The observed plasticity of ectoine content indicates that the associated microbial community contributes substantially to the intracellular ectoine pool and that ectoine content in the xenic cultures can compensate for salinity changes. In contrast, the minor amounts of ectoine in the axenic cultures will not contribute substantially when compared to the other osmolytes present in the cells.

In axenic conditions, homarine together with glycine betaine are the dominant, compatible solutes (Figure 1). Glycine betaine increased under salinity stress (0.5-fold in short term, more than 2-fold in long-term treatments, Figure 2). However, in contrast to ectoine, no influence of the microbial community on the content of this osmolyte was observed. Glycine betaine has been earlier observed as a metabolite compensating for salinity changes in phytoplankton including *T. weissflogii* [2,17]. The independence of the associated microbial community on glycine betaine levels indicates that its regulation in algae is independent of biotic interactions with the associated microbiome, but rather depends on the external salinity.

The third dominant osmolyte is homarine (Figure 1). Homarine has not been described as osmolyte in diatoms before, but in the prasinophyte *Platymonas subordiformis*, as well as in the coccolithophore *E. huxleyi* [2,28]. In xenic cultures, homarine concentrations were substantially lower compared to ectoine concentrations but both were in the pmol cell^−1^ range at 35 PSU salinity (Figure 2). At 35 PSU, homarine production under axenic conditions nearly doubled compared to xenic cultures, indicating a predominant or exclusive algal origin of this metabolite. The elevated amount in axenic cultures is potentially compensating for their overall lower ectoine content. Homarine in xenic cultures increases 3.9-fold after short-term and 3.5-fold after long-term salinity stress. In axenic conditions, a 1.5-fold increase due to short-term salinity stress was observed, while long-term stress had no significant impact on the concentration of this metabolite (Figure 2). In contrast to the ectoine concentration that is substantially dependent on bacterial presence at both salinities, intracellular concentrations of homarine at high salinity are similar in xenic and axenic cultures. Under both axenic and xenic conditions, the response to homarine is already at its maximum after short-term stress and seems to be a fast response in comparison to ectoine and glycine betaine that are both building up during prolonged culturing under high salinity.

### 2.3. Ectoine Uptake by T. weissflogii

**Figure 3:**
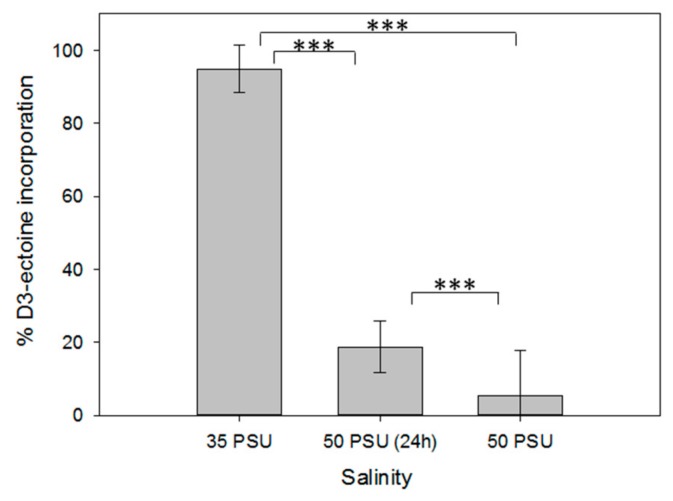
Uptake of stable isotope labeled D_3_-ectoine in axenic cultures of *Thalassiosira weissflogii* CCMP1336 under different salinity conditions (see legend of Figure 2 for explanation of the respective treatments). Error bars represent standard deviation (biological replicates, *N* = 4). Statistical analysis is based on One-Way ANOVA with a Tukey Test for multiple comparison procedures. *** *P* ≤ 0.001.

Due to the difference of intracellular compatible solute concentration between xenic and axenic cultures, we investigated whether microalgae can take up extracellular ectoine that might be provided by associated bacteria. Therefore, we incubated axenic cultures of *T. weissflogii* cultivated in artificial seawater of different salinities, supplied with 1 µM of stable isotope (deuterium) labeled D_3_-ectoine. We determined the relative amount of the labeled metabolite in relation to the cellular ectoine by integrating the respective ions of the labeled and unlabeled form (Figure 3). Under all salinity conditions, labeled ectoine exceeded that of the unlabeled form, indicating a substantial uptake of the externally supplied substrate. The relative amount of labeled ectoine in comparison to the unlabeled metabolite was maximal at 35 PSU, the salinity at which the lowest cellular content was observed in Figure 2B. With increasing salinity, the proportion of ectoine taken up in its labeled form decreased, which is mainly due to the increased content of the unlabeled metabolite (Figure 2 and Figure 3). According to these results, the higher content of ectoine in xenic cultures of *T. weissflogii* compared to the axenic ones can be explained by uptake of the compound supplied by bacteria. Microalgae can thus act as a sink of this metabolite, which becomes particularly available when environmental changes in salinity occur.

Several studies reported uptake mechanisms for zwitterionic osmolytes in marine algae [8,29]. *T. weissflogii*, a diatom that is not producing DMSP, relies entirely on the uptake of DMSP to fulfill cellular functions [7]. Our results demonstrate that this diatom also takes up ectoine very efficiently. This suggests that *T. weissflogii* generally uses external zwitterionic metabolites to counteract osmotic stress. Two transport systems for compatible solutes (ATP-Binding Cassette Transporters (ABC Transporters) and Betaine-Choline-Carnitine Transporters (BCCT)) have been reported in marine micro-organisms. They recognize DMSP and structurally related compounds like glycine betaine and transport them into the cells [7,8,29,30,31]. Likewise, in Gram-positive and Gram-negative bacteria, transport systems for compatible solutes are part of the strategy used by microbial cells to overcome stress. Often there are several systems involved in the uptake of these osmolytes in order to provide a robust strategy to counter the adverse condition [32]. Several ectoine transport systems involved in adaptation to osmotic stress, temperature, and nutrient stress conditions were characterized in bacteria and assigned to four different transport families: (i) binding protein-dependent ABC transporters, (ii) major facilitator family (MFS), (iii) BCCT, and (iv) periplasmatic binding protein-dependent tripartite ATP independent periplasmatic transporter family (TRAP-T) [33,34]. Generally, transport systems have a broad specificity, being able to transport other compatible solutes; but there are also cases, like the TeaABC transporter system in *Halomonas elongata* [35] and the EctT transporter in *Virgibacillus pantothenticus* [36], where the system is specific for ectoine. However, no detailed information on related systems is available for diatoms.

### 2.4. Ectoine in Phaeodactylum tricornutum

We also detected ectoine in the model diatom *Phaeodactylum tricornutum*. In contrast to *T. weissflogii* and most other diatoms, *P. tricornutum* is capable of adjusting its cell size according to the physicochemical conditions in the environment. We were thus interested in the question to which degree the diatom adapts to changes in salinity through adjustment of the cellular ectoine content and its cell size.

**Figure 4:**
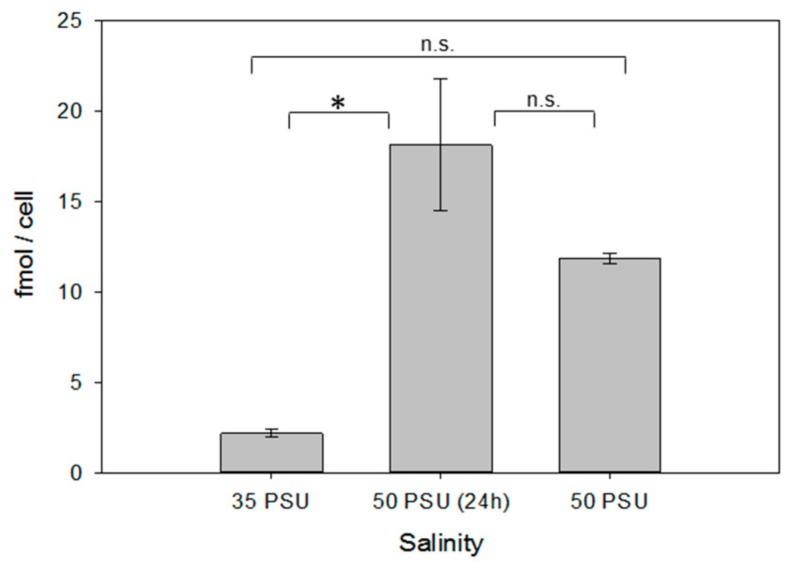
Ectoine content in xenic cultures of *P. tricornutum* under different salinity conditions (see legend of Figure 2 for explanation of the respective treatments). Error bars represent standard deviation (biological replicates, *N* = 4). Statistical analysis is based on One-Way ANOVA with a Tukey Test for multiple comparison procedures. * *P* < 0.05.

As in *T. weissflogii*, in xenic cultures of *P. tricornutum*, increased salinity resulted in significantly elevated cellular ectoine concentrations (Figure 4). Counterintuitively, the cell size increased with increasing salinity by 20.6 % in the long-term salinity stress treatment, compared to the control (*P* < 0.01, Table 1). Thus, there is no adaptation by decreased cell volume and consequently, higher concentrations of cellular metabolites, as it is observed in plant cells. Compensation with acquired ectoine is thus a means of adaptation in this diatom as well.

### 2.5. Ectoine in Other Microalgae

Data of a survey of zwitterionic osmolytes [4] could be used to re-evaluate the distribution of ectoine. Integration of the ectoine signals in the chromatograms revealed that ectoine was always detected in LC-MS in amounts similar to those of DMSP (Table 2). This substantial amount of a novel compatible solute, in diatoms (*T. weissflogii*, *Skeletonema costatum*, and *Phaeodactylum tricornutum*), a dinoflagellate (*Prorocentrum minimum*), two haptophytes (*Prymnesium parvum* and *Isochrysis galbana*), and a coccolithophore (*Emiliania huxleyi*), suggests a massive contribution to overall carbon fluxes in the oceans. Given the turnover of DMSP of ca. 10^9^ tons annually [37,38] and the similar ectoine content, we suggest a massive contribution of this metabolite to nitrogen shuttling within the plankton. Together with DMSP, glycine betaine, and homarine, it clearly belongs to the class of major osmolytes according to the classification in Gebser et al. [2].

## 3. Materials and Methods

### 3.1. Cultivation of Microalgae

Xenic and axenic cultures of *Thalassiosira weissflogii* (RCC76, Roscoff Culture Collection, Roscoff, France; CCMP 1336, Provasoli-Guillard National Center for *Marine* Algae and Microbiota, East Boothbay, ME, USA) as well as a culture of *Phaeodactylum tricornutum* (SCCAP K-1280, http://www.sccap.dk) were cultivated in artificial seawater medium according to Maier and Calenberg [39]. Standing cultures in 50 mL polystyrene cell culture bottles with membrane filter screw caps for gas exchange were maintained at a temperature of 14 °C ± 2 °C. A 14:10 light–dark cycle with light was provided by Osram biolux lamps (40 µmol photons m^−2^s^−1^ between 400 and 700 nm). Cultures were grown to the exponential phase (determined in independent preliminary experiments recording cell counts), diluted 20-fold with fresh medium, and cultivated again to the exponential phase before the analysis. Their axenity was checked during the growth and before the extraction by microscopy and by plating aliquots of each culture on marine broth-agar plates.

### 3.2. Salinity Treatment

For long-term salinity stress tests, cultures of *T. weissflogii* and *P. tricornutum* were grown in artificial seawater with a standard salinity of 35 Practical Salinity Units (PSU), as well as in artificial sea water where the salinity was adjusted, by addition of NaCl, to reach a final concentration of 50 PSU. For short-term salinity stress test, cultures of diatoms were grown in 35 PSU artificial seawater to the exponential phase and 24 h before the extraction, 5 mL of a 2.65 M solution of NaCl was added after sterile filtration to 35 mL cultures, in order to reach a final salinity of 50 PSU. All media were autoclaved before use. For each salinity, microalgae were cultivated in triplicates.

### 3.3. Cell Counting and Size Measurement

To determine the final cell densities, 50 µL of diatoms cultures were analyzed using a BD Accuri™ C6 flow cytometer, with standard filters. The discriminator was set to forward light scatter and samples were analyzed with a flow rate of 35 µL/min. Prior to data collection, the instrument was validated using diluted beads solutions with a known concentration. Pictures for cell size determination were taken with a Leica DFC280 microscope using a Nikon DS-U3 camera. Pictures of 50 randomly selected cells for every salinity were evaluated. Measurements were performed with NIS-Elements Viewer Ver4.50.00. Calculations of the average cell volumes were based on a rectangular shape for *T. weissflogii* and on an ellipsoid shape for *P. tricornutum* [40]. To determine the efficiency of the GF/C filtration to remove bacteria from algal cells (see Section 3.4), 30 mL of a xenic culture of *T. weissflogii* was split. One 15 mL aliquot was diluted with 45 mL of sterile artificial seawater, while the other 15 mL were filtered on Whatman GF/C filters as described below. Filters were washed three times with 15 mL of sterile artificial seawater and the filtered medium was collected. Samples of the filtered medium (250 µL) and of the diluted culture (250 µL) were fixed with glutaraldehyde (1% final concentration) for 15 min and stained with SYBR GOLD^®^ (SYBR™ Gold Nucleic Acid Gel Stain (10,000× Concentrate in DMSO), Thermo Scientific, 10,000-fold diluted from stock solution) for 15 min in the dark. Then, the samples were measured with a BD AccuriTM C6 flow cytometer with the following settings: 25 µL per sample, 4 µL/min, 3 washing steps between each sample with milliQ water.

### 3.4. Sample Preparation

Diatom cells were extracted at the late exponential phase of growth, by filtration of 30 mL of each culture under reduced pressure (Whatman GF/C grade microfiber filters) of 500 mbar, followed by vacuum filtration of 90 mL of sea water. In xenic cultures, this procedure allowed to remove most bacteria as verified by flow cytometry. Filters were immediately transferred into 4 mL glass vials containing 500 µL of methanol, while another 500 µL of methanol was added directly on the filter. After 30 min at room temperature, samples were stored at −20 °C. For ultra-high-pressure liquid chromatography high-resolution mass spectrometry (UHPLC-HRMS) analysis, 50 µL of each extract were diluted with 100 µL of a mixture of acetonitrile and water (9:1 *v*/*v*). After centrifugation (5 min, 4.500× *g*), the supernatant was submitted to UHPLC-HRMS analysis.

### 3.5. Equipment

Analytical separation and quantification were performed on a Dionex Ultimate 3000 system (Thermo Scientific) coupled to a Q-Exactive Plus Orbitrap mass spectrometer (Thermo Scientific). Electrospray ionization was performed in positive mode ionization, with the following parameters: capillary temperature, 380 °C; spray voltage, 3.000 V; sheath gas flow, 60 arbitrary units; and aux gas flow, 20 arbitrary units. The LC separation column was a SeQuant ZIC-HILIC column (5 µm, 2.1 × 150 mm, SeQuant), equipped with a SeQuant ZIC-HILIC guard column (5 µm, 2.1 × 20 mm). NMR spectra were recorded on a Bruker Avance 400 MHz instrument.

### 3.6. Osmolyte Analysis

For separation of osmolytes, the eluent consisted of high-purity water with 2% acetonitrile and 0.1% formic acid (solvent A) and 90% acetonitrile with 10% water and 5 mmol L^−1^ ammonium acetate (solvent B) [11]. The flow rate was set to 0.6 mL min^−1^ and a linear gradient was used for separation with 100% solvent B (2 min), 60% B (11 min), 20% B (11.8 min), 20% B (14.9 min), 100% B (15 min), 100% B (18 min). The column was kept at 25 °C; the injection volume was 2 µL. Identification of ectoine was carried out by addition of ectoine (Sigma-Aldrich) as internal standard; for a proper quantification, a calibration curve of the standard followed by comparisons of the peak area of the analytes with the peak area of the internal standard was performed. The calibration curve (*n* = 3) for the area of the molecular ion was *y* = 7.58 × 10^7^*x* with *r* = 0.9964, limit of detection (LOD) = 0.90 nM, limit of quantification (LOQ) = 2.7 nM. For homarine, the calibration curve (*n* = 3) for the areas of the molecular ion was *y* = 1.29 × 10^8^*x* with *r* = 0.9915, limit of detection (LOD) = 4.96 nM, limit of quantification (LOQ) = 15.03 nM; for glycine betaine, *y* = 1.54 × 10^8^*x* with *r* = 0.9495, limit of detection (LOD) = 0.28 µM, limit of quantification (LOQ) = 0.85 µM.

### 3.7. Uptake of Labeled Ectoine by Marine Diatoms

For determination of ectoine uptake, 40-mL cultures of *T. weissflogii* supplied with 1 µM of D_3_-ectoine were prepared, four replicates per each salinity treatment. Cultures were gravity-filtered on Whatman GF/C during the late exponential phase of growth, and cells on the filter were washed three times with 20 mL of the respective medium. Filters were immediately transferred into 1 mL of methanol. Samples were frozen and further analyzed as described above. During separation, H/D isotope exchange was observed and mass spectrometric data were corrected for the scrambling using data from a run of the pure labeled compound.

### 3.8. Homology Search

Homologues of the enzymes of the ectoine pathway from *Halorhodospira halochloris* were identified by BLAST searches of the *P. tricornutum* genome at NCBI (http://www.ncbi.nlm.nih.gov/sites/ genome) using default parameters.

### 3.9. Synthesis of D3-ectoine

All reactions were performed under an argon atmosphere. All solvents and chemicals were used without further purification. Dry solvents were obtained from VWR (ethanol, electronic grade) and Acros (diethylether (Et_2_O), extra dry, over molecular sieve, AcroSeal^®^). d,l-diaminobutyric acid was bought from Sigma-Aldrich, (Germany) and D_3_-labeled acetonitrile from Euriso-Top (Cambridge Isotope Laboratories).

**Synthesis of D_3_-ethyl acetimidate** [41,42]: Dry HCl gas was bubbled through 5 mL (85.6 mmol) of dry ethanol over a period of 3 h at room temperature. The HCl gas was generated in an argon-purged round-bottom flask by slow dropwise addition of concentrated H_2_SO_4_ onto solid NaCl. The gas outlet of the flask was connected to a drying line filled with CaSO_4_ and introduced to the reaction flask through a syringe needle. Under positive argon pressure, the reaction flask was placed in a dry ice/acetone bath and 5.0 mL (95.7 mmol) of D_3_-labeled acetonitrile was added dropwise. The reaction was brought to 0 °C and stirred overnight. A white solid precipitate formed and dry Et_2_O (12.5 mL) was added. The resulting slurry was stirred for 15–30 min; after this time, the solvent was removed with a syringe and the product was dried under argon flow to give (**1**) (3.48 g, 27.6 mmol, 43%) as a white solid. ^1^H NMR (400 MHz, CDCl_3_) δ ppm: 1.44 (s, 3H, CH_3_), 4.56 (s, -OCH_2_-), 11.19–12.08 (br d, 2H, NH_2_); ^13^C NMR (100 MHz, CDCl_3_) δ ppm: 13.63 (CH_3_), 70.69 (-OCH_2_-), 176.77 (-C=NH_2_).

**Synthesis of D_3_-ectoine:** In a flask, 3.4 g of D_3_-ethyl acetimidate was dissolved in ethanol (30 mL). Under a stream of argon, 6.0 g (30 mmol) of d,l-diaminobutyric acid was added and the reaction was stirred overnight at room temperature. Then, the mixture was heated to reflux for two hours and cooled to 0 °C. After centrifugation, Et_2_O (10 mL) was added to the supernatant and the product precipitated. The solvent was decanted and the precipitate redissolved in ethanol (500 µL). Precipitation with Et_2_O was repeated four times to yield **2** (148 mg, 1.01 mmol, 2%) as a white solid. ^1^H NMR (400 MHz, CDCl_3_): δ 2.24 (m, 2H, C5 CH_2_), 3.45 (m, 2H, C6 CH_2_), 4.36 (t, 1H, C4 CH); ^13^C NMR (100 MHz, CDCl_3_): δ 16.7 (m, CD_3_), 20.51 (C5), 36.73 (C6), 50.66 (C4), 161.09 (C2), 170.87 (C7). ESI-MS (positive) *m/z* 146.100 [M + H]^+^.

## 4. Conclusions

We describe here the prevalence of a novel phytoplankton osmolyte, ectoine, that is found in all investigated marine microalgae. Cellular ectoine content in *T. weissflogii* and *P. tricornutum* is substantially elevated in the presence of bacteria. The axenic algae also produce minor amounts of the compounds, indicating both algae and bacteria as producers. Externally applied ectoine is readily taken up by the algae in amounts sufficient to compensate for osmotic stress.

## Figures and Tables

**Table 1:** Cell size and volume of *T. weissflogii* and *P. tricornutum* under the different salinity conditions.

	Lenght (µm)	Width (µm)	Cell Volume (µm^3^)	Standard Deviation	Difference (Δ%)	One Way ANOVA
***T. weissflogii***						
35 PSU	188	95.1	1.32 × 10^6^	±2.59 × 10^5^		
50 PSU (24 h)	227	85.7	1.23 × 10^6^	±1.74 × 10^5^	−6.82%	n.s.
50 PSU	207	88.9	1.29 × 10^6^	±3.12 × 10^5^	−2.27%	n.s.
***P. tricornutum***						
35 PSU	21.6	11.8	131	±39.1		
50 PSU (24 h)	20.7	10.4	113	±29	−13.70%	n.s.
50 PSU	22.4	13.6	159	±33.8	21.40%	p < 0.01

**Table 2:** Quantitative survey of ectoine production by xenic marine microalgae compared to other zwitterionic metabolites. Chromatograms for ectoine evaluation and values of dimethylsulfoniopropionate (DMSP) and dimethylsulfoxonium propionate (DMSOP) are obtained from Thume et al. 2018 [4]. Replicates: *N* = 3, error bars are based on standard deviation.

Species	GBT	DMSA	Gonyol	*n* DMSP (fmol per cell)	*n* DMSOP (fmol per cell)	*n* Ectoine (fmol per cell)
*P. minimum*	+	+	+	304.5 ± 61.2	3.66 ± 1.23	141.67 ± 13.43
*P. parvum*	+	-	+	16.2 ± 4.4	0.029 ± 0.005	7.85 ± 1.61
*S. costatum*	+	-	-	6.56 ± 2.06	0.029 ± 0.005	5.42 ± 1.89
*E. huxleyi*	+	-	+	4.83 + 0.57	0.029 ± 0.013	2.26 ± 0.45
*I. galbana*	+	-	+	4.69 + 0.27	0.017 ± 0.003	2.62 ± 0.53

## References

[1] Moran M.A., Durham B.P. (2019). Sulfur metabolites in the pelagic ocean. Nat. Rev. Microbiol..

[2] Gebser B., Pohnert G. (2013). Synchronized regulation of different zwitterionic metabolites in the osmoadaption of phytoplankton. Mar. Drugs.

[3] Yoch D.C. (2002). Dimethylsulfoniopropionate: Its sources, role in the marine food web, and biological degradation to dimethylsulfide. Appl. Environ. Microbiol..

[4] Thume K., Gebser B., Chen L., Meyer N., Kieber D.J., Pohnert G. (2018). The metabolite dimethylsulfoxonium propionate extends the marine organosulfur cycle. Nature.

[5] Curson A.R.J., Todd J.D., Sullivan M.J., Johnston A.W.B. (2011). Catabolism of dimethylsulphoniopropionate: Microorganisms, enzymes and genes. Nat. Rev. Microbiol..

[6] Smyth K., Elliott M., Solan M., Whiteley N. (2016). Effects of changing salinity on the ecology of the marine environment. Stressors in the Marine Environment.

[7] Spielmeyer A., Gebser B., Pohnert G. (2011). Investigations of the uptake of dimethylsulfoniopropionate by phytoplankton. ChemBioChem.

[8] Dickschat J.S., Rabe P., Citron C.A. (2015). The chemical biology of dimethylsulfoniopropionate. Org. Biomol. Chem..

[9] Keller M.D., Kiene R.P., Matrai P.A., Bellows W.K. (1999). Production of glycine betaine and dimethylsulphoniopropionate in marine phytoplankton. II. N-limited chemostat cultures. Mar. Biol..

[10] Spielmeyer A., Pohnert G. (2010). Direct quantification of dimethylsulfoniopropionate (DMSP) with hydrophilic interaction liquid chromatography/mass spectrometry. J. Chromatogr. B.

[11] Spielmeyer A., Gebser B., Pohnert G. (2011). Dimethylsulfide sources from microalgae: Improvement and application of a derivatization-based method for the determination of dimethylsulfoniopropionate and other zwitterionic osmolytes in phytoplankton. Mar. Chem..

[12] Zhao P.P., Gu W., Wu S., Huang A., He L., Xie X., Gao S., Zhang B., Niu J., Lin A.P. (2014). Silicon enhances the growth of *Phaeodactylum tricornutum* Bohlin under green light and low temperature. Sci. Rep..

[13] Bussard A., Corre E., Hubas C., Duvernois-Berthet E., Le Corguille G., Jourdren L., Coulpier F., Claquin P., Lopez P.J. (2017). Physiological adjustments and transcriptome reprogramming are involved in the acclimation to salinity gradients in diatoms. Environ. Microbiol..

[14] Garcia N., Lopez-Elias J.A., Miranda A., Martinez-Porchas M., Huerta N., Garcia A. (2012). Effect of salinity on growth and chemical composition of the diatom *Thalassiosira weissflogii* at three culture phases. Lat. Am. J. Aquat. Res..

[15] Ito T., Asano Y., Tanaka Y., Takabe T. (2011). Regulation of biosynthesis of dimethylsulfoniopropionate and its uptake in sterile mutant of *Ulva pertusa* (Chlorophyta). J. Phycol..

[16] Takagi M., Karseno, Yoshida T. (2006). Effect of salt concentration on intracellular accumulation of lipids and triacylglyceride in marine microalgae *Dunaliella cells*. J. Biosci. Bioeng..

[17] Keller M.D., Kiene R.P., Matrai P.A., Bellows W.K. (1999). Production of glycine betaine and dimethylsulphoniopropionate in marine phytoplankton. I. Batch cultures. Mar. Biol..

[18] Dickson D.M.J., Kirst G.O. (1986). The role of b-dimethylsulphoniopropionate, glycine betaine and homarine in the osmoacclimation of *Platymonas subcordiformis*. Planta.

[19] Galinski E.A., Pfeiffer H.-P., Trüper H.G. (1985). 1,4,5,6-Tetrahydro-2-methyl-4-pyrimidinecarboxylic acid. A novel cyclic amino acid from halophilic phototrophic bacteria of the genus *Ectothiorhodospira*. Eur. J. Biochem..

[20] Kolp S., Pietsch M., Galinski E.A., Gutschow M. (2006). Compatible solutes as protectants for zymogens against proteolysis. Biochim. Biophys. Acta.

[21] Kunte H.J., Lentzen G., Galinski E.A. (2014). Industrial production of the cell protectant ectoine: Protection mechanisms, processes and products. Curr. Biotechnol..

[22] Waditee-Sirisattha R., Kageyama H., Takabe T. (2016). Halophilic microorganism resources and their applications in industrial and environmental biotechnology. AIMS Microbiol..

[23] Weinisch L., Kuhner S., Roth R., Grimm M., Roth T., Netz D.J.A., Pierik A.J., Filker S. (2018). Identification of osmoadaptive strategies in the halophile, heterotrophic ciliate *Schmidingerothrix salinarum*. PLoS Biol..

[24] Weinisch L., Kirchner I., Grimm M., Kuhner S., Pierik J.J., Rossello-Mora R., Filker S. (2019). Glycine betaine and ectoine are the major compatible solutes used by four different halophilic heterotrophic ciliates. Microb. Ecol..

[25] Harding T., Roger A.J., Simpson A.G.B. (2017). Adaptations to high salt in a halophilic protist: Differential expression and gene acquisitions through duplications and gene transfers. Front. Microbiol..

[26] Widderich N., Czech L., Elling F.J., Könneke M., Stöveken N., Pittelkow M., Riclea R., Dickschat J.S., Heider J., Bremer E. (2016). Strangers in the archaeal world: Osmostress-responsive biosynthesis of ectoine and hydroxyectoine by the marine thaumarchaeon *Nitrosopumilus maritimus*. Environ. Microbiol..

[27] Landa M., Burns A.S., Roth S.J., Moran M.A. (2017). Bacterial transcriptome remodeling during sequential co-culture with a marine dinoflagellate and diatom. ISME J..

[28] Dickson D.M.J., Kirst G.O. (1987). Osmotic adjustment in marine eukaryotic algae—The role of inorganic-ions, quaternary ammonium, tertiary sulfonium and carbohydrate solutes. I. Diatoms and a Rhodophyte. New Phytol..

[29] Kiene R.P., Williams L.P.H., Walker J.E. (1998). Seawater microorganisms have a high affinity glycine betaine uptake system which also recognizes dimethylsulfoniopropionate. Aquat. Microb. Ecol..

[30] Van Bergeijk S.A., Van der Zee C., Stal L.J. (2003). Uptake and excretion of dimethylsulphoniopropionate is driven by salinity changes in the marine benthic diatom *Cylindrotheca closterium*. Eur. J. Phycol..

[31] Torstensson A., Young J.N., Carlson L.T., Ingalls A.E., Deming J.W. (2019). Use of exogenous glycine betaine and its precursor choline as osmoprotectants in Antarctic sea-ice diatoms. J. Phycol..

[32] Czech L., Bremer E. (2018). With a pinch of extra salt—Did predatory protists steal genes from their food?. PLoS Biol..

[33] Onraedt A., De Mey M., Walcarius B., Soetaert W., Vandamme E.J. (2006). Transport kinetics of ectoine, an osmolyte produced by *Brevibacterium epidermis*. Biotechnol. Lett..

[34] Czech L., Hermann L., Stoveken N., Richter A.A., Hoppner A., Smits S.H.J., Heider J., Bremer E. (2018). Role of the extremolytes ectoine and hydroxyectoine as stress protectants and nutrients: Genetics, phylogenomics, biochemistry, and structural analysis. Genes.

[35] Grammann K., Volke A., Kunte H.J. (2002). New type of osmoregulated solute transporter identified in halophilic members of the *Bacteria* domain: TRAP transporter TeaABC mediates uptake of ectoine and hydroxyectoine in *Halomonas elongata* DSM 2581T. J. Bacteriol..

[36] Kuhlmann A.U., Hoffmann T., Bursy J., Jebbar M., Bremer E. (2011). Ectoine and hydroxyectoine as protectants against osmotic and cold stress: Uptake through the SigB-controlled betaine-choline- carnitine transporter-type carrier EctT from *Virgibacillus pantothenticus*. J. Bacteriol..

[37] Kiene R.P., Linn L.J., Bruton J.A. (2000). New and important roles for DMSP in marine microbial communities. J. Sea Res..

[38] Simó R. (2001). Production of atmospheric sulfur by oceanic plankton: Biogeochemical, ecological and evolutionary links. Trends Ecol. Evolut..

[39] Maier I., Calenberg M. (1994). Effect of extracellular Ca^2+^ and Ca^2+^-antagonists on the movement and chemoorientation of male gametes of *Ectocarpus siliculosus* (Phaeophyceae). Bot. Acta.

[40] Hillebrand H., Durselen C.D., Kirschtel D., Pollingher U., Zohary T. (1999). Biovolume calculation for pelagic and benthic microalgae. J. Phycol..

[41] Himdi-Kabbab S., Lavrador K., Bazureau J.P., Hamelin J. (1995). Synthesis of 1,4,5,6-Tetrahydro 2-Methyl 4-Pyrimidine Carboxylic Acid: Osmoprotector Amino Acid. Synth. Commun..

[42] Shortreed M.R., Lamos S.M., Frey B.L., Phillips M.F., Patel M., Belshaw P.J., Smith L.M. (2006). Ionizable isotopic labeling reagent for relative quantification of amine metabolites by mass spectrometry. Anal. Chem..

